# Pattern recognition receptor signaling in otitis media: immune crosstalk and pathogenic mechanisms

**DOI:** 10.3389/fimmu.2026.1804078

**Published:** 2026-04-28

**Authors:** Mingwen Guo, Shaoyan Zhang, Zhencheng Liao, Daqing Yan, Xinyuan Tan, Chunling Liu

**Affiliations:** Department of Otorhinolaryngology Head and Neck Surgery, The People’s Hospital of Baoan, Shenzhen, China

**Keywords:** immunity, inflammasome, inflammation, NLRP3, otitis media, pattern recognition receptors, toll-like receptors

## Abstract

Otitis media (OM) remains a prevalent and multifactorial inflammatory disease of the middle ear, especially in pediatric populations. The immune system, particularly pattern recognition receptors (PRRs), plays a central role in detecting microbial pathogens and initiating host defenses. TLR2 and TLR4 mediate bacterial clearance but exhibit subtype-specific dysregulation in chronic OM forms. NOD1, NOD2, and NLRP3 modulate intracellular pathogen sensing and inflammasome activation, while RIG-I governs antiviral immunity. C-type lectin receptors (CLRs) are emerging as modulators of both innate and adaptive responses, yet their mechanistic roles remain insufficiently explored. Cross-talk among PRRs and immune evasion by microbial biofilms contribute to chronicity and recurrence. Understanding these interactions and age- or genotype-related PRR variations may inform precision immunotherapies. This review summarizes current understanding of Toll-like receptors (TLRs), nucleotide-binding oligomerization domain-like receptors (NLRs), retinoic acid–inducible gene I-like receptors (RLRs), and CLRs in OM pathophysiology. By elucidating the PRR-mediated signaling landscape, this review highlights the intricate PRR-mediated signaling landscape in OM, offering insights into potential therapeutic targets and future translational strategies for improving OM management.

## 1 Introduction

Otitis media (OM) encompasses a heterogeneous group of middle ear conditions. Clinically, it is categorized into otitis media with effusion (OME), suppurative otitis media, middle ear cholesteatoma, and other specific subtypes. Suppurative otitis media is further stratified into acute (AOM) and chronic suppurative otitis media (CSOM) (1, 2). OM frequently arises from polymicrobial infections; in addition to bacterial agents, several major respiratory viruses, including rhinovirus, respiratory syncytial virus, adenovirus, and influenza A, are closely associated with AOM onset. Disruption of innate immune mechanisms within the middle ear contributes to sustained inflammation and impairs microbial clearance, thereby facilitating chronic and recurrent OM (3, 4). Within the middle ear mucosa, pattern recognition receptors (PRRs) serve as primary sensors for a wide array of microbial threats (5, 6). These receptors are classified according to their subcellular distribution into membrane-associated and intracellular categories. The membrane-bound group includes TLRs and CLRs, while cytosolic PRRs encompass NLRs and RLRs, each mediating distinct signaling pathways in host defense (7). Innate defense is mediated through PRRs, strategically localized across extracellular, membrane-associated, and cytosolic domains, allowing for precise detection of non-self molecular motifs. These receptors recognize pathogen-associated molecular patterns (PAMPs) from bacterial or viral origin and trigger intracellular signaling cascades, including the activation of MAPKs, NF-κB, and stress-responsive kinases (8, 9). The resulting signaling events culminate in the production of proinflammatory mediators, cytokines, interferons, and chemokines, that collectively enhance immune surveillance and microbial clearance. In otitis media, these responses manifest as epithelial thickening, macrophage infiltration, and accumulation of effusion fluid (10).

Importantly, the consequences of PRR dysregulation are not uniform across OM subtypes. In OME, inadequate or blunted PRR signaling may preferentially promote epithelial secretory reprogramming rather than efficient pathogen eradication, thereby favoring EGFR-associated goblet cell metaplasia, SPDEF/MUC5AC-driven mucin accumulation, and reduced FOXJ1-dependent ciliogenesis, all of which impair mucociliary clearance and contribute to persistent effusion (5, 11–13). By contrast, in CSOM, prolonged exposure to biofilm-derived ligands is more likely to sustain maladaptive PRR activation, resulting in disruption of epithelial junctional proteins such as claudins and ZO-1, induction of matrix-remodeling enzymes including MMPs, and chronic mucosal damage that supports suppuration, bacterial persistence, and irreversible tissue remodeling (6, 14). These subtype-specific differences suggest that PRR signaling in OM is not merely a trigger of inflammation, but also a determinant of whether the middle ear mucosa evolves toward fluid retention and remodeling or toward destructive chronic infection (15). This review highlights the immunological functions of specific PRR subsets in OM, emphasizing their mechanistic roles in disease initiation and progression.

## 2 Toll-like receptors in otitis media

**Figure 1:**
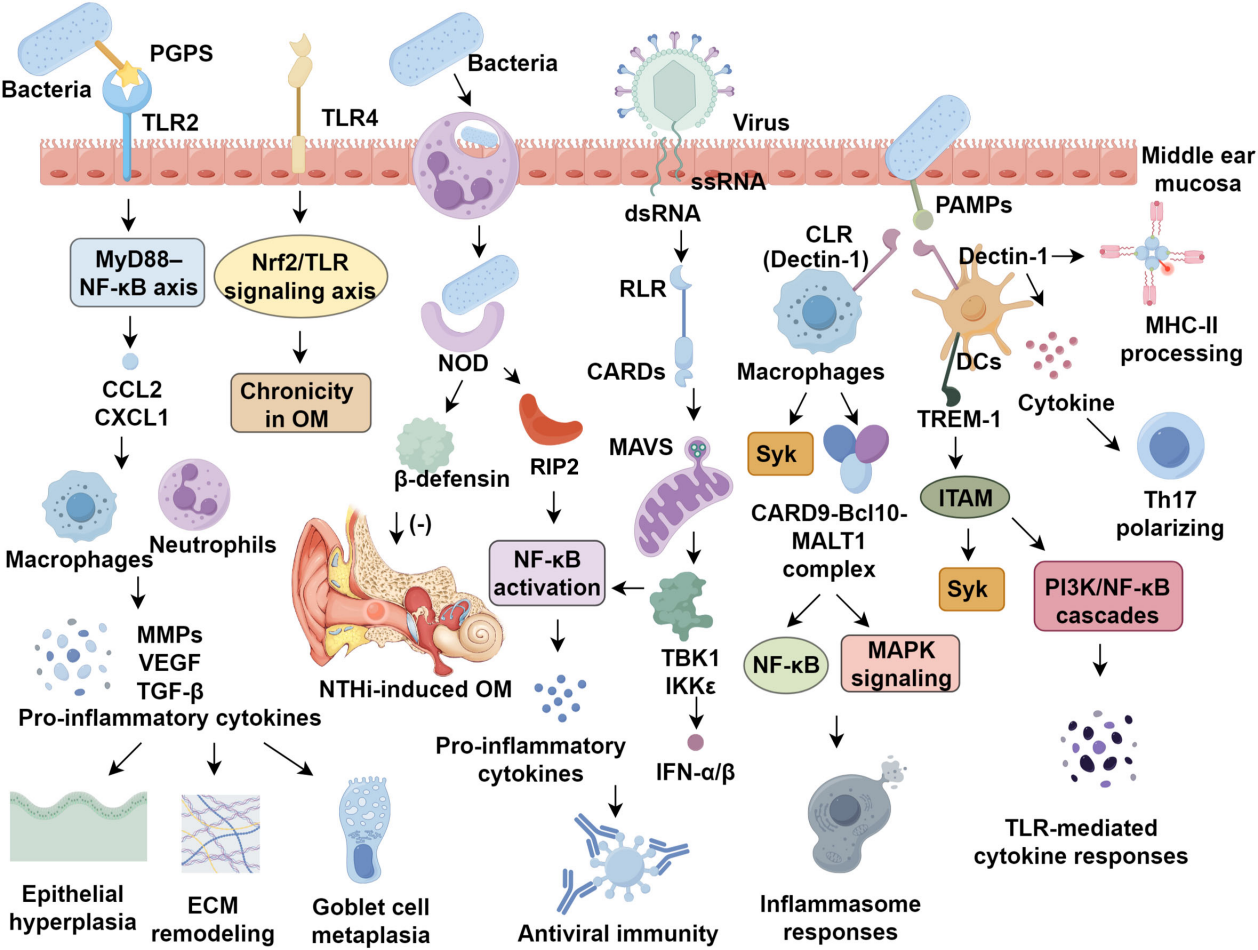
Pattern recognition receptors orchestrate immune system in otitis media.

PRRs are distributed throughout the middle ear mucosa and act as key sensors for detecting diverse classes of pathogens. Based on their cellular localization, PRRs are classified into membrane-bound and cytoplasmic types (16). Membrane-bound PRRs include TLRs and CLRs, whereas cytoplasmic PRRs comprise NLRs and RLRs (17). Distinct TLRs are localized to specific cellular compartments and recognize different PAMPs or damage-associated molecular patterns (DAMPs) (18). TLR1, TLR2, TLR4, TLR5, and TLR6 are located on the cell surface, where they detect extracellular PAMPs: TLR1 and TLR2 recognize triacylated lipoproteins and peptidoglycan (PGPS); TLR2 and TLR6 detect diacylated lipoproteins and lipoproteins (19); TLR4 senses lipopolysaccharides (LPS); and TLR5 detects flagellin. Conversely, TLR3, TLR7, TLR8, and TLR9 reside in intracellular vesicles, enabling recognition of intracellular PAMPs: TLR3 detects viral double-stranded RNA (20); TLR7 and TLR8 recognize single-stranded RNA with viral nucleic acid-like structures; and TLR9 senses unmethylated CpG DNA released within endosomes after bacterial degradation by macrophages and other immune cells (21). Of these, TLR2 and TLR4 are most closely associated with OM pathogenesis. Upon activation by microbial ligands, TLRs primarily signal through MyD88 and TRIF (22, 23). The MyD88-dependent pathway predominantly activates NF-κB, leading to the transcription and release of pro-inflammatory cytokines and chemokines, whereas the TRIF-dependent pathway induces type I interferon production and activation (24). Disruption of any component in these signaling cascades, including TLRs, MyD88, TRIF, or NF-Κb, can result in dysregulated immune responses in the middle ear (25, 26) (**Figure 1**).

### 2.1 TLR2 in otitis media

Clinical and experimental evidence highlights a pivotal role for TLR2 in OM pathogenesis. Immunohistochemical analyses of mucosal tissues from patients with OM and cholesteatoma reveal elevated TLR2 expression relative to healthy controls, suggesting TLR2-mediated regulation of cytokines, chemokines, interferons, and defensins may drive inflammation (27). In contrast, middle ear fluid (MEF) from pediatric OME patients exhibits reduced TLR2 mRNA levels, implicating deficient TLR2 expression in OME susceptibility (28). Similarly, markedly diminished TLR2 expression in CSOM patients may underlie persistent inflammation and impaired bacterial clearance (13). Differential TLR2 expression across OM subtypes likely reflects distinct pathogenic mechanisms and effector cell recruitment patterns (29). Kaur et al. (30) further demonstrated elevated TLR2 mRNA in culture-positive samples, particularly in polymicrobial infections, and genetic variants of TLR2 have been associated with recurrent AOM. TLR2 signals predominantly through the MyD88-dependent pathway, recruiting IRAK4, IRAK1, TRAF6, and TAK1 to activate NF-κB and MAPK cascades (p38, JNK), thereby inducing rapid transcription of proinflammatory mediators including TNF, IL-1β, IL-6, CXCL2, and CCL3 (31, 32). This signaling module is particularly adept at establishing an early chemokine gradient that recruits neutrophils and monocytes to the infected middle ear, facilitating bacterial phagocytosis and resolution of acute infection (10, 33). Consistent with this paradigm, MyD88-deficient murine models of OM exhibit delayed leukocyte recruitment, impaired macrophage-mediated killing of nontypeable Haemophilus influenzae (NTHi), and prolonged bacterial persistence, underscoring the centrality of MyD88-coupled TLR2 signaling in early antibacterial defense (22, 34).

However, chronic exposure to bacterial ligands, particularly within biofilm-embedded communities, may induce a tolerance-like state characterized by attenuated TLR2 responsiveness (35, 36). This adaptive desensitization involves upregulation of negative regulators including IRAK-M, Tollip, A20 (TNFAIP3), and SOCS1, which disrupt proximal signaling by limiting MyD88–IRAK1/4–TRAF6 complex assembly (13). Concurrently, chronic stimulation enhances miR-146a expression, which post-transcriptionally suppresses IRAK1 and TRAF6, further dampening inflammatory output despite persistent pathogen presence (37, 38). In TLR2^⁻/⁻^ mice, PGPS challenge results in exacerbated OM characterized by heightened inflammation, goblet cell depletion, impaired ciliary function, and increased mortality compared to wild-type counterparts (39). TLR2 activation triggers the MyD88–NF-κB axis, inducing transcription of chemokines such as CCL2 and CXCL1, which recruit macrophages and neutrophils to the inflamed mucosa (40, 41). These infiltrating cells facilitate pathogen clearance and secrete MMPs, VEGF, TGF-β, and pro-inflammatory cytokines, promoting epithelial hyperplasia, goblet cell metaplasia, and extracellular matrix (ECM) remodeling, hallmarks of OM pathology (12, 42). The absence of TLR2 delays epithelial recovery, perpetuating inflammation and mucosal damage. Consistently, TLR2^⁻/⁻^ mice upon *Streptococcus pneumoniae* infection, underscoring the receptor’s essential role in bacterial clearance and resolution of inflammation (33). These findings indicate TLR2 as a central regulator of innate immune responses in OM and a promising therapeutic target for enhancing bacterial clearance and attenuating chronic disease progression.

### 2.2 TLR4 in otitis media

Clinical investigations consistently report altered TLR4 expression in OM patient cohorts, though directionality varies by disease phenotype and anatomical compartment. In both OME and CSOM, TLR4 protein and mRNA levels in middle ear mucosa or effusion samples are frequently reduced relative to healthy controls, suggesting that insufficient TLR4-mediated surveillance may compromise bacterial clearance and predispose to disease chronicity (43). Peripheral blood immune cells from children with recurrent AOM and healthy controls reveal the elevated TLR2 and TLR4 expression in the recurrent AOM cohort. However, whether a definitive link exists between local and systemic inflammatory responses in OM remains contentious (28, 44). Notably, mRNA levels of nuclear factor erythroid 2–related factor 2 (Nrf2), TLR2, and TLR4 in MEF from CSOM patients were found to be higher than those from OME patients, suggesting that the Nrf2/TLR signaling axis may drive chronicity in OM, impairing recovery after middle ear tissue injury and facilitating the transition from AOM to COM (45).

TLR4 signaling is uniquely characterized by its capacity to engage MyD88 and TRIF. TLR4 preferentially recruits the MyD88-dependent cascade via TIRAP/Mal, activating IRAK4, IRAK1, TRAF6, and TAK1 to drive rapid NF-κB and MAPK (p38/JNK) nuclear translocation (46–49). This early-phase signaling induces robust production of TNF, IL-1β, IL-6, CXCL2, and CCL3, establishing a steep chemokine gradient that recruits neutrophils and initiates bacterial clearance (50–53). Following receptor endocytosis, TLR4 transitions to a TRIF-dependent signaling mode via TRAM, culminating in IRF3 activation and type I interferon (IFN-α/β) production (48, 54, 55). This late-phase response enhances antiviral defenses, promotes antigen presentation, and modulates resolution pathways. In OM, MyD88-skewed signaling dominates acute bacterial eradication, whereas TRIF-dependent outputs may influence chronic inflammation and tissue remodeling (34). TLR4-deficient mice exhibit earlier inflammatory peaks but impaired bacterial clearance compared to TLR2-deficient counterparts, suggesting that TLR4 governs initial pathogen recognition while TLR2 sustains eradication efforts (56).

TLR4 facilitates neutrophil extracellular trap (NET) formation during acute OM by regulating reactive oxygen species (ROS) production and autophagic flux in bone marrow–derived (57) neutrophils challenged with Streptococcus pneumoniae (58). This TLR4–NET axis enhances extracellular bacterial trapping and clearance, providing a mechanistic link between receptor signaling and innate effector function. Additionally, during the CSOM, an interaction between TLR4 and Nrf2 has been identified; TLR4 deficiency coupled with Nrf2 overexpression collectively exacerbates chronic disease development (59). Furthermore, persistent TLR4 stimulation by biofilm-derived LPS may induce tolerance-like states through upregulation of negative regulators (IRAK-M, SOCS1), paralleling mechanisms described for TLR2 (35, 57). Such adaptive desensitization, while potentially protective against immunopathology, may inadvertently permit bacterial persistence and recurrent inflammation.

## 3 Nucleotide-binding oligomerization domain-like receptors in otitis media

### 3.1 NOD1 and NOD2 in otitis media

Clinical transcriptomic profiling reveals that NOD1 and NOD2 expression in middle ear tissues follows a non-linear, age-dependent trajectory, which contribute to differences in the severity of OM-associated inflammation (60, 61). Upon ligand recognition, NOD1 and NOD2 undergo conformational changes that expose their NOD domains, enabling oligomerization and recruitment of the serine/threonine kinase RIP2 (RICK) via CARD–CARD interactions (62, 63). The NOD–RIP2 complex subsequently activates TAK1 and IKK complexes, culminating in NF-κB nuclear translocation and MAPK (p38, JNK, ERK) phosphorylation. This canonical cascade drives transcription of proinflammatory cytokines, chemokines, and crucially, epithelial-derived antimicrobial peptides (AMPs) (64–66). In nontypeable *Haemophilus influenzae* (NTHi)-induced OM, NOD1^⁻/⁻^ and NOD2^⁻/⁻^ mice exhibit markedly delayed bacterial clearance, reduced immune cell infiltration, and sustained middle ear inflammation compared to wild-type controls (67). NOD2 signaling has been directly linked to the induction of β-defensin 2 (BD-2), a cationic AMP with potent bactericidal and chemotactic properties (68). In the absence of functional NOD2, BD-2 production is blunted, compromising epithelial barrier integrity and diminishing early microbial containment. These findings establish NOD1/NOD2 not merely as inflammatory triggers, but as essential coordinators of mucosal antimicrobial programming and inflammatory resolution in the middle ear.

Beyond altered receptor expression, host PRR gene variants may contribute to susceptibility to recurrent AOM. Variants in TLR2 may weaken ligand-driven TLR2/TLR1 or TLR2/TLR6 heterodimer signaling, thereby reducing the efficiency of bacterial lipoprotein recognition and blunting downstream induction of epithelial antimicrobial programs (50, 69, 70). Likewise, TLR4 polymorphisms are proposed to impair the assembly or signaling competence of the TLR4–MD-2–CD14 receptor complex, which may attenuate early LPS sensing, limit neutrophil- and macrophage-coordinated bacterial clearance, and prolong middle-ear inflammation (71, 72). NOD2 variants may compromise RIP2-dependent signaling, with downstream effects on β-defensin production, epithelial barrier reinforcement, and mucosal immune priming, thereby creating a permissive niche for pathogen persistence and recurrent disease (73, 74).

### 3.2 NLRP3 in otitis media

NLRP3 inflammasome assembly requires two temporally and functionally distinct signals. Signal 1 (priming) is predominantly mediated by TLRs, IL-1 receptors, or TNF receptors engaging NF-κB, which transcriptionally upregulates NLRP3, pro-IL-1β, and pro-IL-18 (75, 76). This priming step establishes a sensitized state within middle ear epithelial cells and resident macrophages. Signal 2 (activation) is triggered by diverse PAMPs or DAMPs, inducing conformational changes that promote NLRP3 oligomerization, recruitment of the adaptor protein ASC, and subsequent activation of caspase-1 (77–79). Active caspase-1 cleaves pro-IL-1β and pro-IL-18 into their mature, biologically active forms, which are then secreted via gasdermin D (GSDMD)-mediated pyroptotic pores or non-classical secretory pathways (80, 81). In human OM, this paradigm is clinically substantiated. NLRP3 mRNA and protein expression demonstrated are elevated in cholesteatoma and chronic OM (COM) mucosa compared to healthy controls, correlating with increased IL-1β and IL-18 levels in middle ear effusions (82). These findings position NLRP3 inflammasome activity as a hallmark of chronic middle ear pathology rather than an acute defense mechanism.

In COM, these triggers may include extracellular ATP-mediated P2X7 receptor activation, which promotes K^+^ efflux and facilitates inflammasome assembly; intracellular Ca^2+^ disequilibrium; and mitochondrial dysfunction, which increases mitochondrial reactive oxygen species (mtROS) and promotes dissociation of thioredoxin-interacting protein (TXNIP), enabling its interaction with NLRP3 (83–85). Chronic accumulation of keratin debris, cholesterol-rich material, and damaged cellular components may destabilize lysosomes, resulting in cathepsin B release, another recognized driver of NLRP3 activation (86). At the level of inflammasome assembly, NEK7 may function as a licensing factor downstream of ionic flux, stabilizing the NLRP3 complex and facilitating ASC recruitment and caspase-1 activation (87, 88). Collectively, these mechanisms suggest that in COM NLRP3 activation is not merely a passive consequence of infection, but rather the product of a broader pathogenic network in which TLR-dependent transcriptional priming, ionic perturbation, oxidative mitochondrial stress, and lysosomal injury converge to sustain IL-1β/IL-18 maturation, epithelial remodeling, and chronic tissue-destructive inflammation.

## 4 RLRs and CLRs in otitis media

### 4.1 RLRs in otitis media

RLRs, a class of cytoplasmic pattern recognition receptors analogous to NLRs, comprise RIG-I, MDA5, and LGP2, which detect viral RNA during infection (89). Under homeostatic conditions, RLRs are expressed at low levels and remain inactive. Upon recognition of 5’-triphosphate–bearing short dsRNA or ssRNA from RNA viruses, RIG-I undergoes conformational activation, exposing its CARD domains to engage the mitochondrial adaptor MAVS (90–92). This interaction initiates downstream signaling through TBK1 and IKKϵ, leading to IRF3/IRF7 phosphorylation and nuclear translocation, culminating in type I interferon (IFN-α/β) production (93–95). Concurrent activation of NF-κB promotes pro-inflammatory cytokine release. In AOM, where respiratory viruses frequently precede bacterial colonization, RIG-I–mediated type I IFN signaling is essential for early antiviral responses, maintaining epithelial barrier integrity, and priming innate defenses (96). Impaired IFN responses may exacerbate bacterial superinfection and prolong inflammation. Although RLR signaling is central to antiviral immunity and immune-mediated disease (97), studies in OM remain limited. In a pediatric study, Kim et al. reported significantly reduced mRNA expression of TLR9, NOD1, and RIG-I in OME-susceptible children relative to controls, implicating RIG-I as a protective factor against recurrent OM (98). Current OM-related RLR research is largely confined to RIG-I; functional roles of MDA5 and LGP2 remain to be elucidated.

### 4.2 CLRs in otitis media

C-type lectin receptors (CLRs) represent a structurally diverse pattern recognition receptor family that senses pathogen-associated glycan motifs and modulates both innate and adaptive immunity. Expressed predominantly on macrophages and dendritic cells, CLRs such as Dectin-1, DC-SIGN, and TREM-1 recognize fungal, bacterial, and endogenous glycoconjugates, triggering Syk kinase–dependent signaling via ITAM-bearing adaptors or ITAM-like motifs (99–102). This cascade recruits the CARD9–BCL10–MALT1 complex, which cooperates with TLR pathways to fine-tune NF-κB activation intensity, duration, and transcriptional bias (103–105). In otitis media, CLR–TLR crosstalk likely shapes inflammatory outcomes beyond canonical single-receptor models. For instance, simultaneous CLR and TLR engagement may lower the activation threshold for proinflammatory cytokine production (TNF, IL-1β, IL-6), amplifying early antibacterial responses (101, 106, 107). Conversely, DC-SIGN–dependent Raf-1 signaling can qualitatively reprogram NF-κB activity by promoting p65/RelA post-translational modifications, thereby altering promoter selectivity and prolonging expression of specific resolution-associated genes (108, 109).

TREM-1, a myeloid amplifier signaling through DAP12/Syk/PI3K, synergizes with TLRs to augment IKK activation and sustain inflammatory outputs, a mechanism that may contribute to chronic mucosal remodeling when dysregulated (110, 111). In the biofilm-rich middle ear microenvironment, such signal integration could determine whether inflammation resolves efficiently or progresses toward persistent disease (6, 50). Clinical evidence for CLRs in OM remains sparse. Two transcriptomic studies reported altered CLR mRNA expression in OME, COM, and cholesteatoma versus controls, suggesting involvement in disease chronicity (112). However, these investigations were limited to transcriptional profiling; protein-level validation, cellular localization, and functional characterization are lacking. Consequently, the mechanistic contribution of specific CLR subtypes to OM pathogenesis, particularly their roles in biofilm recognition, antigen presentation, and T-cell priming, remains undefined.

## 5 Conclusion

Otitis media (OM) is increasingly understood as a disorder of dysregulated mucosal immunity rather than a simple consequence of microbial infection. At the center of this process lies a highly interconnected pattern recognition receptor (PRR) network, in which TLRs, NLRs, RLRs, and CLRs collectively govern the detection of pathogens, the calibration of inflammatory intensity, and the balance between microbial clearance and tissue preservation. The available evidence indicates that PRR signaling in OM is not uniformly protective: when precisely coordinated, it promotes pathogen elimination and restoration of epithelial homeostasis; when excessive, insufficient, or chronically sustained, it drives mucin hypersecretion, barrier disruption, inflammasome activation, biofilm persistence, and progressive mucosal remodeling. In this framework, OM should be viewed as the outcome of context-dependent immune miscalibration across distinct middle-ear microenvironments.

A major challenge moving forward is to define OM not by single receptors, but by the integrated behavior of receptor networks across disease stages and clinical subtypes. Future work should clarify how PRR crosstalk, signal duration and amplitude, host age, genetic susceptibility, and polymicrobial biofilms shape divergent inflammatory trajectories ranging from self-limited acute disease to refractory chronic pathology. Equally important is the identification of cell-specific and stage-specific immune signatures that can distinguish protective from pathogenic inflammation. Such advances may enable biomarker-guided stratification and support the development of mechanism-based therapies that selectively recalibrate innate immune signaling rather than indiscriminately suppress inflammation. A deeper understanding of the PRR-centered immune regulation of OM will be essential for transforming both the biological interpretation and clinical management of OM.
